# Will the Relaxation of COVID-19 Control Measures Have an Impact on the Chinese Internet-Using Public? Social Media-Based Topic and Sentiment Analysis

**DOI:** 10.3389/ijph.2023.1606074

**Published:** 2023-08-10

**Authors:** Yu Xin, Xiaoshuang Tan, Xiaohui Ren

**Affiliations:** ^1^ Department of Science and Technology, West China Hospital, Sichuan University, Chengdu, Sichuan, China; ^2^ West China School of Public Health and West China Fourth Hospital, Sichuan University, Chengdu, Sichuan, China

**Keywords:** COVID-19, social-media, China, relaxation, control measures, sentiment

## Abstract

**Objective:** In December 2022, the Chinese government announced the further optimization of the implementation of the prevention and control measures of COVID-19. We aimed to assess internet-using public expression and sentiment toward COVID-19 in the relaxation of control measures in China.

**Methods:** We used a user-simulation-like web crawler to collect raw data from Sina-Weibo and then processed the raw data, including the removal of punctuation, stop words, and text segmentation. After performing the above processes, we analyzed the data in two aspects. Firstly, we used the Latent Dirichlet Allocation (LDA) model to analyze the text data and extract the theme. After that, we used sentiment analysis to reveal the sentiment trend and the geographical spatial sentiment distribution.

**Results:** A total of five topics were extracted according to the LDA model, namely, Complete liberalization, Resource supply, Symptom, Knowledge, and Emotional Outlet. Furthermore, sentiment analysis indicates that while the percentages of positive and negative microblogs fluctuate over time, the overall quantity of positive microblogs exceeds that of negative ones. Meanwhile, the geographical dispersion of public sentiment on internet usage exhibits significant regional variations and is subject to multifarious factors such as economic conditions and demographic characteristics.

**Conclusion:** In the face of the relaxation of COVID-19 control measures, although concerns arise among people, they continue to encourage and support each other.

## Introduction

According to the statement made at the fourteenth meeting of the International Health Regulations (2005) Emergency Committee regarding the coronavirus disease (COVID-19) pandemic, COVID-19 still remains a dangerous infectious disease with the capacity to cause substantial damage to health and health systems [1]. Starting as a Public Health Emergency of International Concern (PHEIC), the pandemic has infected the world for more than 3 years. In the face of this highly infectious virus, many countries have implemented several strict policy measures such as a variety of travel restrictions, lockdowns, and physical distancing measures to prevent transmission [2, 3].

The unique characteristics of pandemic virus infection indicate that the public are fearful about the spread of the virus in large populations [4]. This fear is a negative emotion that may cause widespread public fear and cause mental distress at the population level [5, 6]. At the same time, the public also has different responses to the PHEIC. Reflecting on the public health emergency management of COVID-19, the public in many countries believe that the pandemic had clear gaps in its processes for managing responses and resilience to pandemics [7, 8]. The negative public reaction is likely to affect the management of COVID-19. This seems to be the case of Italy, where as public trust in government organizations decreases, citizens protest once the government introduces new protective measures [9]. Therefore, the public response is crucial when they are faced with a PHEIC.

With the rapid development of the Internet, social media is an additional source of information that is widely considered by all groups in the face of emergencies [10, 11]. With the epidemic of COVID-19 and the change of relevant countermeasures, the public hope to vent or share their views on COVID-19 through social media. Simultaneously, the government and official organizations through social media release plenty of information, such as their response to public concerns, health knowledge promotion, and crisis management, to guide people to actively face COVID-19. In previous studies, the main methods of obtaining public responses were through questionnaires and social media [7, 8, 12]. Interestingly, these two methods are comparable [13].

Social media has a broad impact on public sentiment. Hussain et al. studied the attitudes of people residing in the United Kingdom and the United States toward the COVID-19 vaccine through Facebook and Twitter. Through the results of natural language processing and deep learning-based techniques, it was found that the overall positive sentiments of the UK and the US are more than 50% on average, and the public is optimistic about vaccine development, effectiveness, and trials [14]. Murimo et al. using data harvested from Twitter, extracted noteworthy topics including alcohol sale and consumption, staying at home, daily tracing statistics, police brutality, 5G, and vaccines conspiracy theories in September 2020 in South Africa [15]. Han et al. used social media to mine and analyze the relevant public opinion on COVID-19 in China in 2020 and found that there was synchronization between frequent daily discussions on microblogs and the outbreak trend of COVID-19 [13].

As China has a large population and relatively weak *per capita* medical resources, it initially had stricter control over the COVID-19 epidemic than other countries, such as case detection and management, lockdown and intercity travel prohibition, physical distancing, and personal protection [16]. On 7 December 2022, the joint prevention and control mechanism of the State Council of China held a press conference to introduce the further optimization and implementation of the epidemic prevention and control measures, which also marked the relaxation of COVID-19 control measures in China [17]. However, this also means that China will face a significant amount of population infection and large consumption of medical resources, which is a huge challenge for the country. Furthermore, severe acute respiratory syndrome and psychological problems after suffering from COVID-19 have had a tremendous impact on the public [18, 19]. Therefore, there is a contradiction in respect to the above prevention and control measures that is being experienced by the public, which they are eager for freedom, but they are afraid of suffering from COVID-19.

Consequently, this study identified public opinion on the relaxation of COVID-19 prevention and control measures as expressed on social media and used microblog text data from the period from 7 December 2022, to 7 January 2023, to analyze internet-using public expression and sentiment toward COVID-19.

## Methods

### Ethical and Legal Considerations

This study does not fall under the scope of the Ethical Review Methods for Life Sciences and Medical Research involving humans, jointly authorized by the National Health Commission, the Ministry of Education, the Ministry of Science and Technology, and the Administration of Traditional Chinese Medicine in China [20]. According to the above regulation article 32, research that employs publicly available data obtained legally or through non-intrusive observation of public acts, as well as research utilizing anonymized information, may be exempt from ethical review. Thus, we have taken appropriate measures to safeguard the information of the subjects involved in this study, as follows: 1) the Excel files retrieved from Weibo have been completely anonymized (they include only the content and address of the posts); 2) the original dataset has been destroyed. In subsequent analyses, we only used post content and location data. Therefore, the privacy of the people under study will not be disclosed.

### Study Design

**FIGURE 1:**
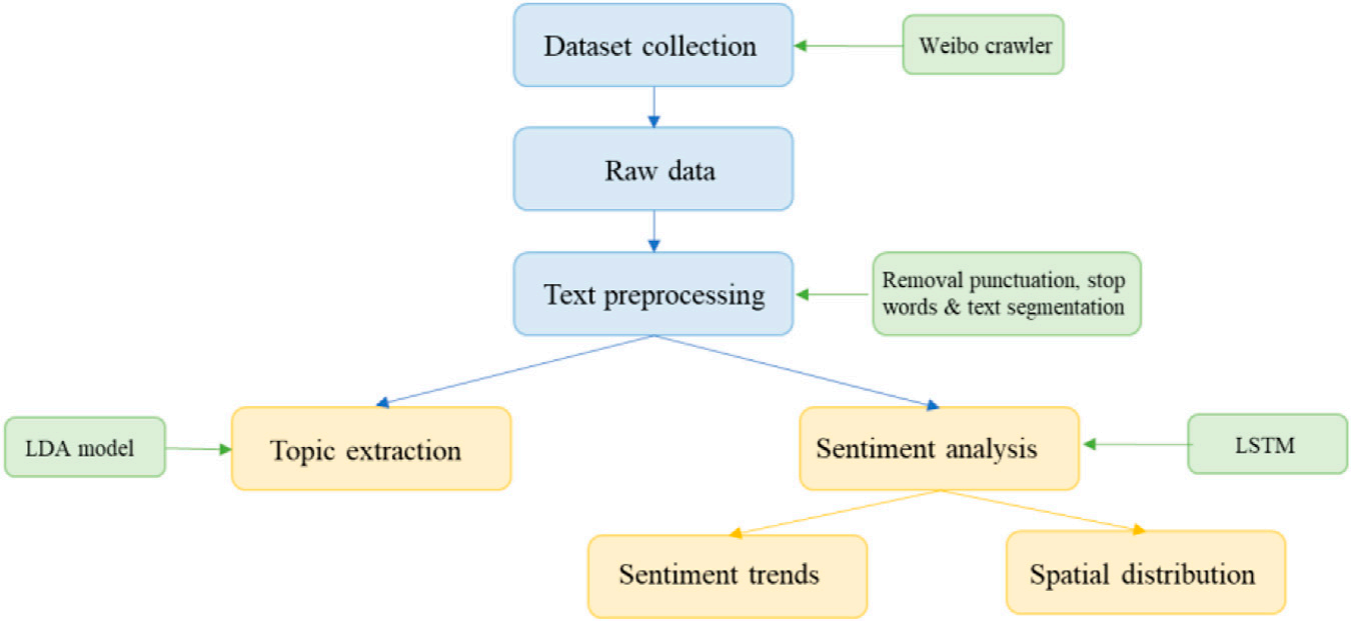
The processes of topic extraction and sentiment classification (China, 2023).

Figure 1 shows the process framework of this study. This study used a user-simulation-like web crawler to collect raw data from Sina-Weibo and then processed the raw data, including the removal of punctuation, stop words, and text segmentation. After performing the above processes, we analyzed the data in two aspects. Firstly, we used the LDA model to analyze the text data and extract the theme, and then we used sentiment analysis to reveal the sentiment trend and the geographical spatial sentiment distribution.

### Data Collection

**FIGURE 2:**
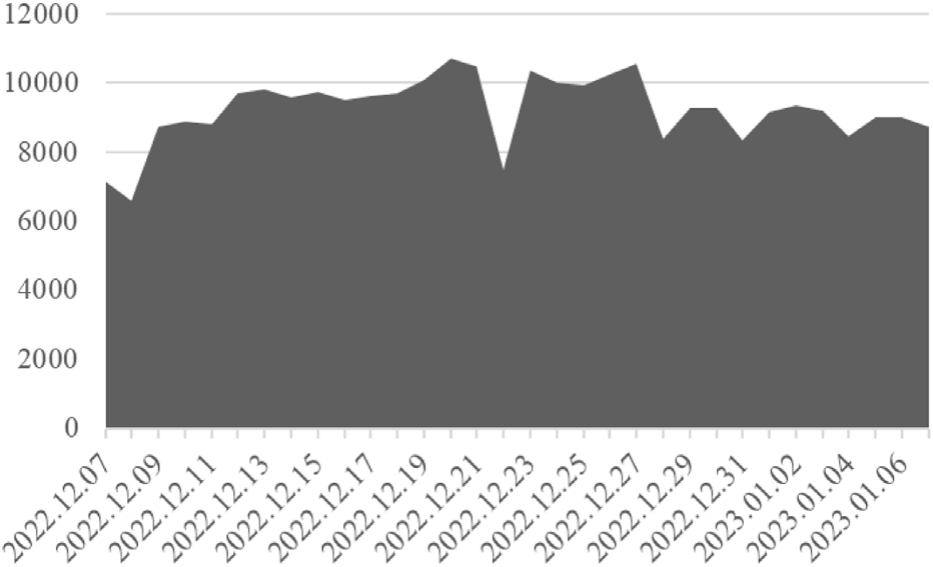
Daily posts on the theme of COVID-19 (China, 2023).

Our data were derived from Sina-Weibo (https://m.weibo.cn), often referred to as Weibo. Weibo is the most popular social platform in China, with 582 million monthly active users. We mainly used the scrappy-based web crawler to obtain content about COVID-19 in Weibo from the period from 07 December 2022, to 07 January 2023. A total of 295,876 pieces of data were obtained, including 8,025 with geographic locations (shown in Figure 2). The data extracted in this study included nickname, text, posting location, and release time by using the “scrapy” package in Python.

### Data Preprocessing

In text analysis, some words without practical meaning need to be deleted to improve the accuracy of the experimental outcomes. In this study, Baidu stop words and HIT stop words were mainly used to remove “meaningless” words from the content obtained from Weibo [13]. After removing the stop words, the next step was to segment the text. Chinese text needs a special word segmentation tool; thus, this study used the “jieba” package in Python to complete the segmentation of Chinese text.

### Topic Analysis

LDA is a kind of unsupervised machine learning technology, known as a three-layer (document–topic–word) Bayesian probability model [21, 22], which can be used to identify hidden topic information in large-scale document sets or corpora. The LDA generation process is as follows: 1) For each document, extract the subject distribution parameters; 2) For each topic, gain from the multi-distribution of the vocabulary on the subject; 3) For certain vocabulary in document, the subject is obtained according to the polynomial distribution [23].

In this study, the “sklearn” package and the “pyLDAvis” package in Python were used. The “sklearn” package was mainly used for topic extraction of the text data, and the “pyLDAvis” package was used to visualize the result of the topic extraction. The amount of topic extraction was mainly based on the perplexity scores. Perplexity scores are the degree to which the model is generated from the corpus [23].

### Sentiment Analysis

**FIGURE 3:**
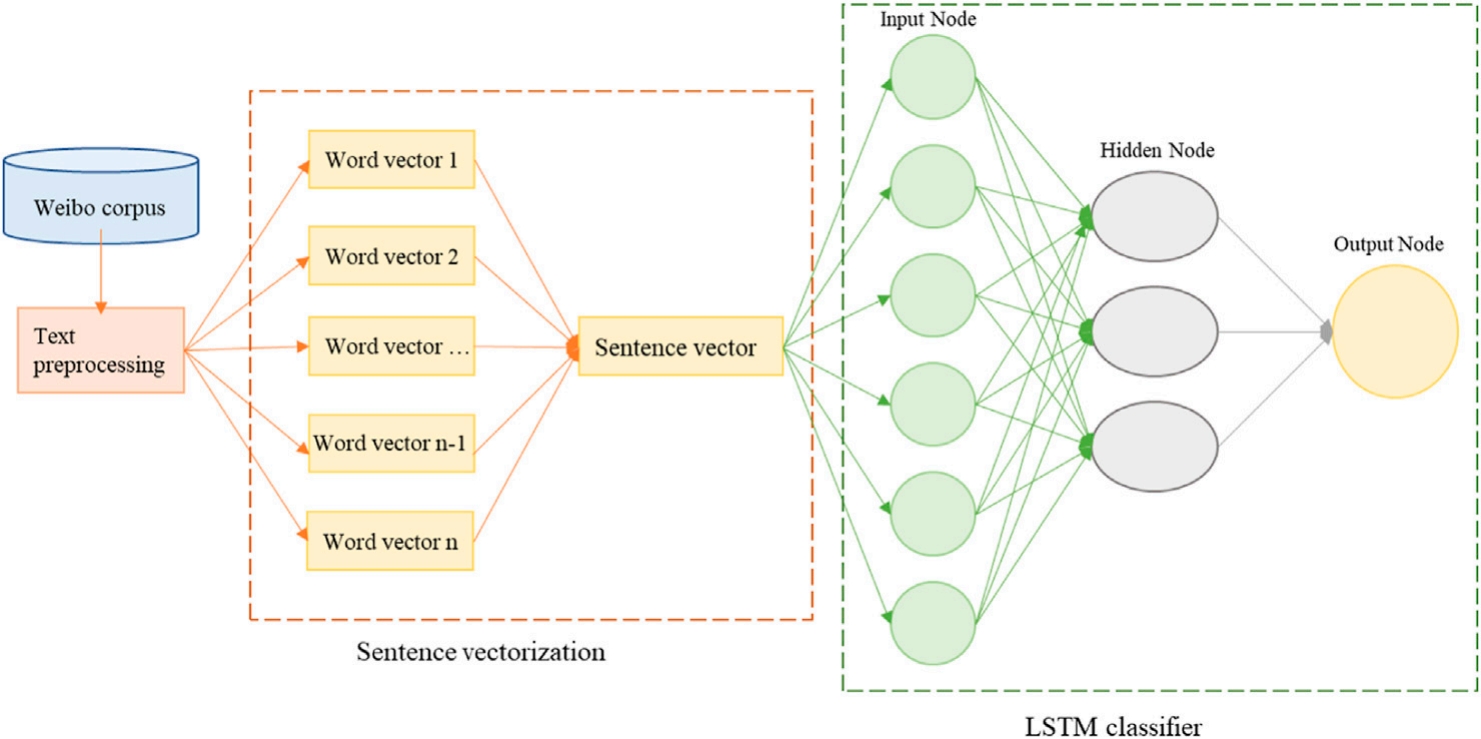
Structure diagram of the COVID-19 weibo sentiment classification model (China, 2023).

The character of Weibo information is that of short texts, which include fewer words and use words more irregularly than traditional long texts. Therefore, LSTM (Long Short-Term Memory) was used in this study for sentiment classification. Compared with the Convolutional Neural Network (CNN), LSTM can consider the semantic association between contexts [24]. The LSTM model built in this study is shown in Figure 3. After text preprocessing, the word vector model was used to represent the semantics of the text as a numeric vector, and then the word vector was used to obtain the sentence vector. The LSTM model consists of three components, including input layer, hidden layer, and output layer, on the basis of which the time information was considered. Subsequently, the model was compiled, trained, evaluated, and applied [25, 26].

The metrics of evaluating performance for LSTM contain accuracy, precision, recall, and F1-score. Accuracy is the proportion of the predicted correct number of the total number of microblogs. Precision, known as positive predictive value, is the proportion of positive/negative microblogs that are correctly predicted by the model over all positive/negative predictions. Recall, known as sensitivity, is the proportion of positive/negative microblogs that are correctly predicted by the model over all positive/negative microblogs. F1-score is a primary metric that combines precision and recall.

## Results

### Topic Modeling

**FIGURE 4:**
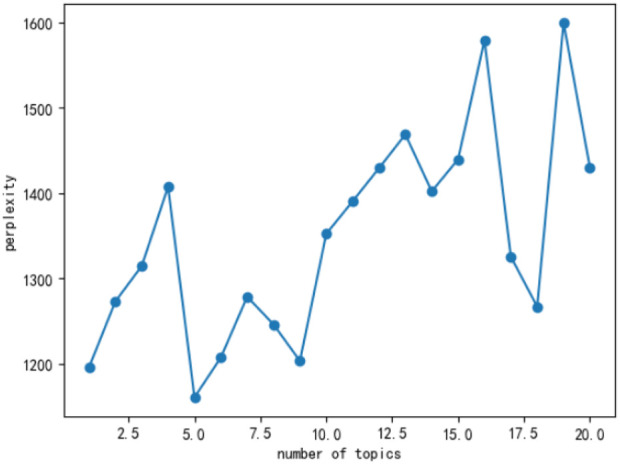
Perplexity scores in a different number of topics (China, 2023).

**FIGURE 5:**
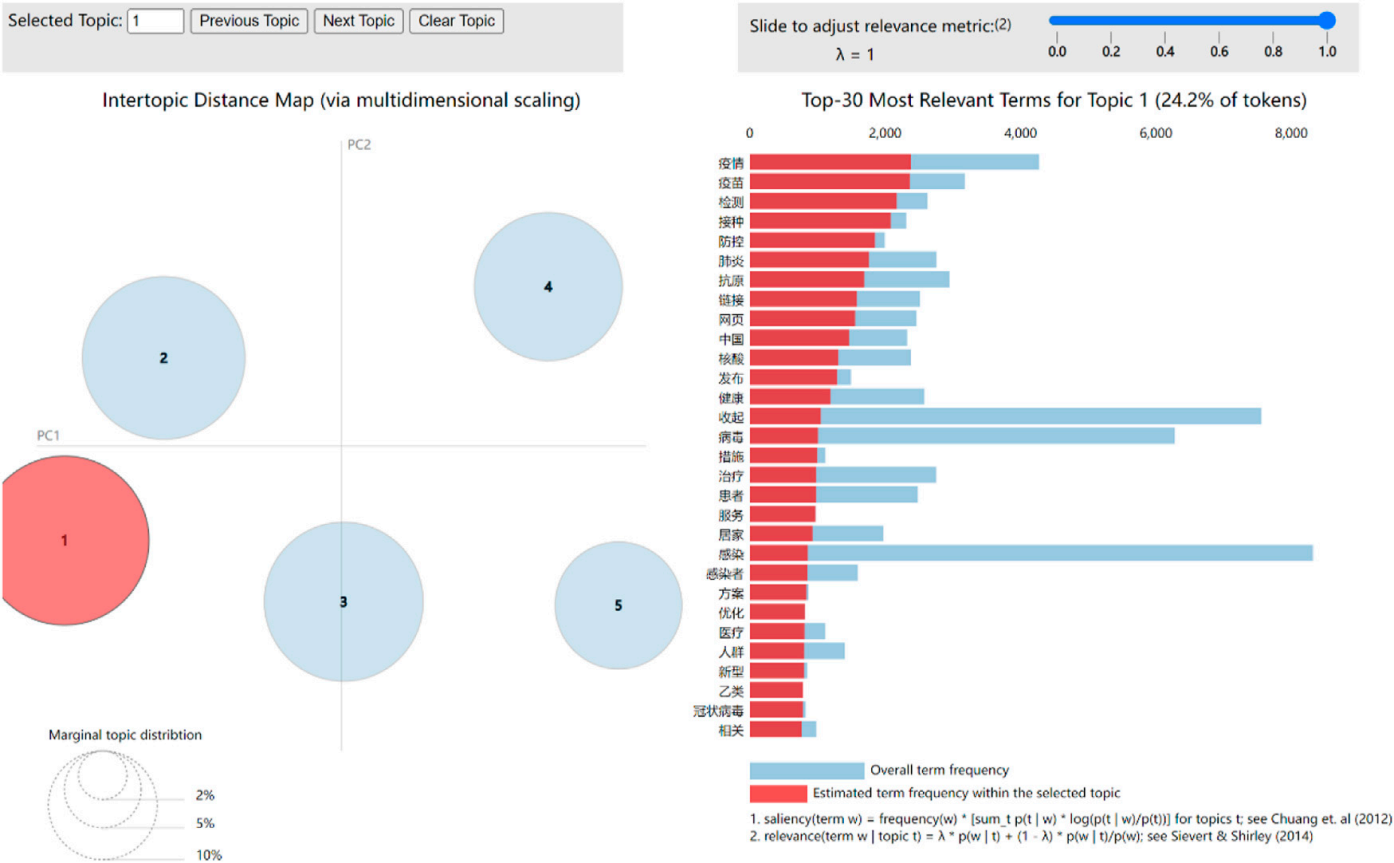
LDA visualization results (China, 2023).

According to Figure 4, the perplexity score is lowest when the number of topics is five. Therefore, five topics were obtained in this study. Figure 5 shows the visualization of the selected LDA model, where the image on the left side shows the distribution of the five topics, and the right side represents the top 30 most highlighted terms of the selected topic. The blue bar shows the overall term frequency in the dataset, and the red bar represents the estimated term frequency in the selected topic.

Table 1 shows the five topics obtained in this study: 1) The 3-year epidemic control has ended, and everyone is witnessing this historic moment in China. 2) After the release from epidemic control, the supplies of nucleic acid antigen, vaccination, treatment, and other medical materials and resources are discussed. 3) Symptoms after infection are mainly systemic, and the most prominent are throat, head, nasal cavity, taste, and other symptoms. 4) Due to the sudden relaxation of COVID-19 control measures in China, domestic authoritative media and hospitals have begun to popularize relevant suggestions for the public. 5) The public, whether sick or not, have all kinds of fears about COVID-19. Therefore, Weibo serves as a platform that offers the public an outlet to express their emotions. This study found that although people are worried about the deregulation of COVID-19 control, they encourage and help each other.

**TABLE 1:** Each topic and topic coding results (China, 2023).

Topic	Topic label	10 representative words selected from the top 20 most salient words
1	Complete liberalization	三年(3 years), 放开(Release), 结束(End),人类(Human), 疫情(epidemic), 希望(Hope), 生活(Life), 感染(Infection), 病毒(Virus), 口罩(Respirator)
2	Resource supply	疫苗(Vaccine), 接种(Inoculation), 防控(Prevention and Control), 抗原(Antigen test), 核酸(Nucleic acid), 健康(Health), 治疗(Treatment), 发布(Announcement), 措施(Measurement), 中国(China)
3	Symptom	感冒(Cold), 症状(Symptom), 嗓子(Throat), 浑身难受(Feel uncomfortable), 头疼(Headache), 味觉(Gustation), 高烧(High fever), 鼻塞(Rhinobyon), 睡不着(Sleepless), 退烧(Coolify)
4	Knowledge	央视网(China Central Television), 专家(Specialist), 医院(Hospital), 医生(Doctor), 康复(Recovery), 患者(Patient), 需要(Need), 建议(Suggestion), 防护(Protection), 用药(Medications)
5	Emotional Outlet	后遗症(Sequela), 难受(Uncomfortable), 大姨妈(Menstruation), 不想(In no mood), 上班(Working), 折磨(Torment), 早日康复(Grow recovery), 妈妈(Mom), 宝宝(Baby), 啊啊啊(Ah, ah, ah)

### The Sentiment Trend in the Sample of “COVID-19 Topic Posts”

The results of the data preprocessing and text segmentation show that 15,7505 microblogs were marked as positive labels, 11,0706 were negative, and the rest of them were marked as neutral. Supplementary File S1 shows the metrics of performance for LSTM. The results show that the accuracy of sentiment analysis in this study was 75%.

Supplementary File S2 shows the ratio of positive and negative microblogs from 7 December 2022, to 7 January 2023. In this Figure, more people expressed positive microblogs than negative. However, there are differences in the trend between the above two lines. Before 21 December 2022, it can be seen that the positive rate had an obvious downward trend, while the negative rate had an obvious upward trend, but the trend was similar after December 21.

### Spatial Distribution of Sentiment Information in the Sample of “COVID-19 Topic Posts”

Excluding non-cities and non-autonomous regions, 7,820 microblogs with the region were obtained in this study, of which the top five cities are shown in Supplementary File S3. Among microblogs with geographical locations, Beijing had the largest number of microblogs, followed by Shanghai, Chengdu, Guangzhou, and Shenzhen.

Supplementary File S4 shows the spatial distribution of sentiment information in the sample of “COVID-19” topic microblogs. Supplementary File S4A shows the distribution of the total number of microblogs. It can be seen from the map that the regions with more microblogs were concentrated in the east and less in the west. To ensure the accuracy of the research, we eliminated cities or autonomous regions with fewer than 10 microblogs in the analysis of positive and negative proportions. However, in Supplementary File S4B, C, this study found that Weibo with a higher positive and negative proportion was more diffuse.

## Discussion

This study has established a topic extraction and classification model to mine the topics and sentiments of COVID-19-related microblogs. After the end of the 3 years of strict epidemic control in China, a large number of microblogs expressed their feelings about it.

As seen from the results of the topic extraction, the five topic labels were Complete liberalization, Resource supply, Symptom, Knowledge, and Emotional Outlet. These five topics are closely related to the relaxation of COVID-19 control measures, mainly reflected in both individual and government aspects. From an individual point of view, with the sudden release from the COVID-19 prevention and control measures, the public rushed to hoard medical supplies, resulting in a shortage of resources in a short period, and at the same time, various symptoms appeared after a large-scale infection, which needed to be shared and vented. Government and official organizations promote the dissemination of positive emotions, indicating that governments can better fulfill their role as crisis communicators by leveraging such media networks [27]. While publicizing through the media, the Chinese government also actively produces medical supplies. China has had different management systems for COVID-19 in different periods. This research focuses on the expression after the release from the COVID-19 management controls. However, Shi et al., who examined Weibo posts from 1 December 2019 to 30 April 2020, found that the sudden outbreak of the COVID-19 epidemic brought “fear” to the public, and their topics focused on traffic measures, materials, and social relationships [28]. Li et al. found that discussion topics among the internet-using public at the beginning of the pandemic included the causative agent of the disease, changing epidemiological characteristics of the outbreak, public reaction to the outbreak control, and response measures [29]. Under the normalization of the COVID-19 epidemic, the topics changed to infection and death caused by the COVID-19 epidemic, control of personnel with positive nucleic acid tests, and persistence of the COVID-19 epidemic, etc. [30]. Therefore, at the different stages of the COVID-19 epidemic, the topics discussed by the internet-using public have been different.

In this study, the LSTM model was used to classify the sentiment of Weibo texts with an accuracy of 75%. The trends of the Weibo positive rate curve and the negative rate curve were found to be different. In the early days of the relaxation of COVID-19 control measures, the internet-using public expressed more positive emotions. It may be that after the end of 3 years of epidemic control, the public felt joyful and excited. The rapid spread of COVID-19 caused widespread infection among the public, resulting in various painful symptoms and more negative emotions [22, 31]. Nonetheless, the proportion of positive sentiment remained higher than that of negative sentiment, which is consistent with the findings of Zhang et al. This may be attributed to the public’s efforts to improve their physical condition by reducing susceptibility and enhancing recovery speed [29, 32, 33]. The spatial distribution results of the sentiment analysis show that the cities with the largest number of microblogs were large cities with large populations, especially Beijing and Shanghai, which may be because the public in large cities tend to use social media to express their thoughts and experiences [34]. The level of microblog activity in the eastern region surpassed that of the western region, which can be potentially attributed to the latter’s superior economic development and greater familiarity with social media for online interaction [35].

Nevertheless, this study has some limitations. First, it was based on text obtained from microblogs, which may introduce selection bias as relevant information cannot be accessed for groups that do not publish online. Second, we only used crawled text information and did not include video and image information. Third, the low proportion of Weibo texts containing location information resulted in the insufficient analysis of spatial distribution characteristics. Despite these limitations, this study contributes to the perspectives of the PHEIC demonstrated on social media by presenting a reliable approach.

### Conclusion

This study used microblog text data from the period of the relaxation of COVID-19 control measures in China. We have proposed a model for topic extraction and sentiment classification, which demonstrates the accuracy and feasibility of these methods in analyzing public sentiment toward health events on the internet.

A total of five topics were extracted according to the LDA model, namely, Complete liberalization, Resource supply, Symptom, Knowledge, and Emotional Outlet. This suggests that social media can sense public perceptions of COVID-19 and can be used as a way to measure internet-using public sentiment. Furthermore, the sentiment analysis indicated that while the percentages of positive and negative microblogs fluctuated over time, the overall quantity of positive microblogs exceeded that of negative ones. Meanwhile, the geographical dispersion of the studied internet-using public exhibited significant regional variations and was subject to multifarious factors such as economic conditions and demographic characteristics.
