# Supplementary material for: Will the Relaxation of COVID-19 Control Measures Have an Impact on the Chinese Internet-Using Public? Social Media-Based Topic and Sentiment Analysis
Source: Int J Public Health. 2023 Aug 10;68:1606074. doi: 10.3389/ijph.2023.1606074 (PMC10448249; doi:10.3389/ijph.2023.1606074)
Supplement: Supplementary file 1 [file DataSheet1.pdf]

Supplementary File 1:

Table Metrics for Evaluating LSTM Performance (China.2023)

	Precision	Recall	F1-score*
Negative	0.78	0.82	0.80
Positive	0.70	0.63	0.66
Accuracy			0.75

F1-score=2 (Precision * Recall)/ (Precision + Recall)

Supplementary File 2:

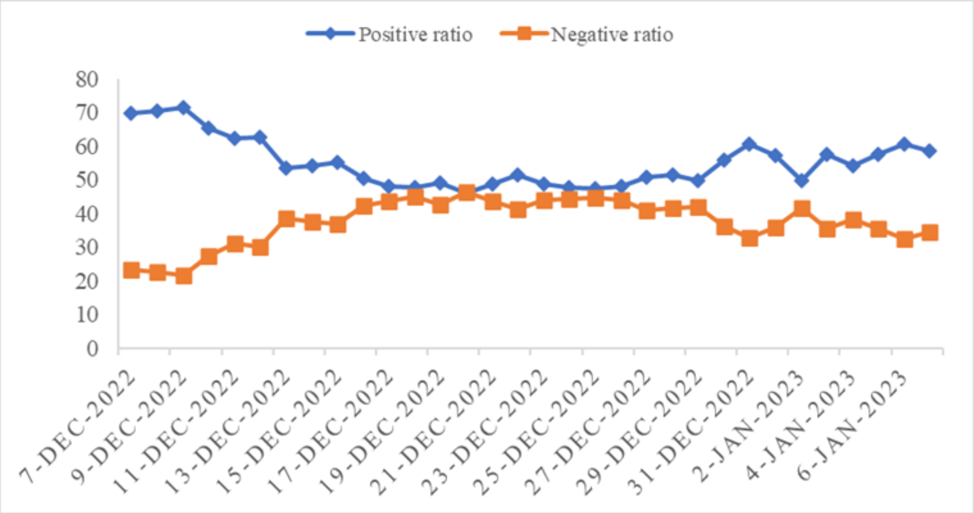

Figure: The ratio of positive and negative microblogs over time (China.2023)

Supplementary File 3:

Table Five cities with the largest number of microblogs (China.2023)

Rank	Province	City	Total posts	Positive Rate (%)	Negative Rate (%)
1	Beijing	Beijing	959	62.4	30.1
2	Shanghai	Shanghai	464	57.5	31.0
3	Sichuan	Chengdu	300	56.3	34.7
4	Guangdong	Guangzhou	296	55.1	34.5
5	Guangdong	Shenzhen	295	54.6	37.3

Supplementary File 4:

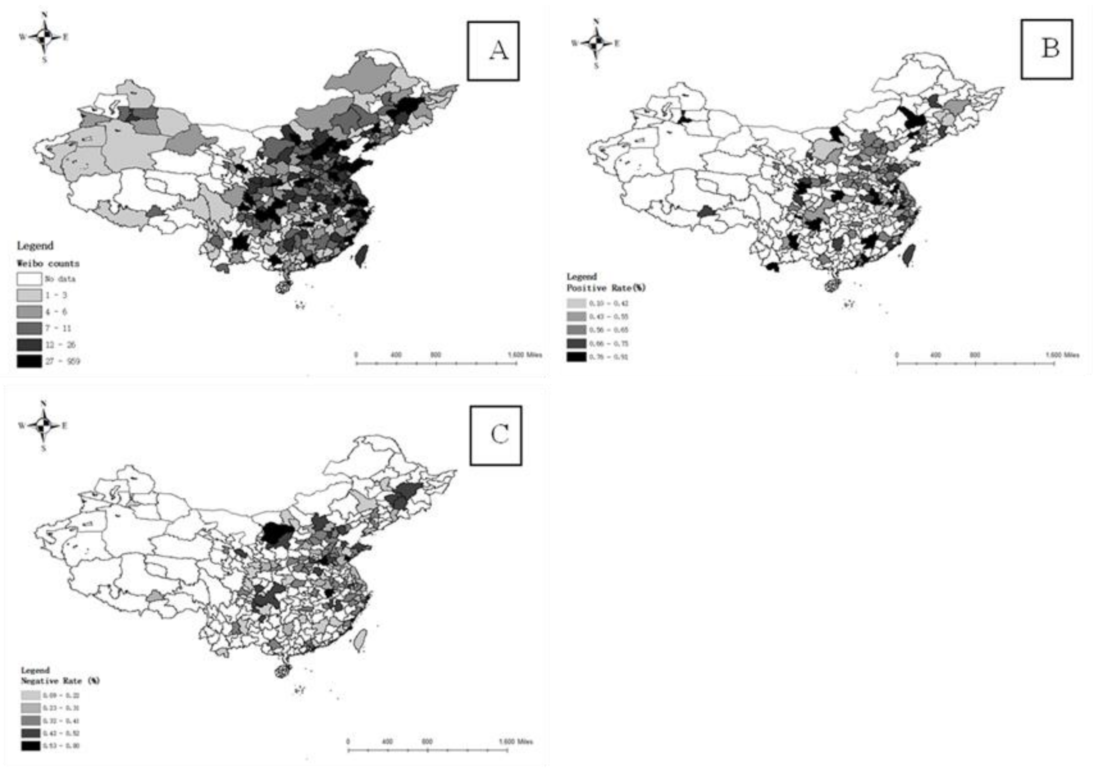

Figure: The spatial distribution of sentiment information in the sample of "COVID-19 topic posts"

A.The spatial distribution of the total number of microblogs; B.The spatial distribution of the positive rate; C.The spatial distribution of the negative rate (China.2023)

Map Source: https://www.tianditu.gov.cn/
